# Report of a case of acinic cell carcinoma of the upper lip and review of Japanese cases of acinic cell carcinoma of the minor salivary glands

**DOI:** 10.4317/jced.53049

**Published:** 2016-12-01

**Authors:** Shigeo Ishikawa, Hitoshi Ishikawa, Shigemi Fuyama, Takehito Kobayashi, Takayoshi Waki, Yukio Taira, Mitsuyoshi Iino

**Affiliations:** 1 Department of Dentistry, Oral and Maxillofacial Plastic and Reconstructive Surgery, Faculty of Medicine, Yamagata University, 2-2-2 Iida-nishi, Yamagata 990-9585, Japan; 2 Yamagata Saisei Hospital, Department of Health Information Management, 79-1 Oki-machi, Yamagata 990-8545, Japan; 3 Department of Diagnostic Pathology, Okitama Public General Hospital, 2000 Nishi-Otsuka, Kawanishi, Higashi-Okitama-gun, Yamagata 992-0601, Japan; 4 Department of Dentistry, Oral and Maxillofacial Surgery, Okitama Public General Hospital, 2000 Nishi-Otsuka, Kawanishi, Higashi-Okitama-gun, Yamagata 992-0601, Japan; 5 Department of Otolaryngology and Head and Neck Surgery, Okitama Public General Hospital, 2000 Nishi-Otsuka, Kawanishi, Higashi-Okitama-gun, Yamagata 992-0601, Japan

## Abstract

Acinic cell carcinoma (ACC) is a malignant tumor of the salivary glands. The majority of ACCs occur in the parotid gland, and ACCs of the minor salivary glands (MSGs) are relatively infrequent. We describe here a patient with ACC of the upper lip. The patient was a 31-year-old male who presented with a nodular mass on the left upper lip. The preoperative diagnosis was benign tumor or cyst, and the lesion was surgically excised. The histological diagnosis was ACC. The postoperative course was uneventful. No recurrence or metastasis was detected at 13 months postoperatively. In addition, we retrospectively reviewed 21 reported Japanese patients with ACC of the MSGs. In 7 of the 21 patients, the preoperative diagnosis was benign tumor, and the tumors were resected without preoperative biopsy. Kaplan–Meier analysis showed that disease-free survival was worse in patients who underwent resection with a preoperative diagnosis of benign tumor than in patients who underwent resection with a preoperative diagnosis of malignant tumor. The rate of recurrence was higher for ACCs assumed to be benign lesions on a purely clinical basis, or without an accurate preoperative biopsy. ACCs of the MSGs are easy to be misdiagnosed for benign lesions such as mucous cysts or hemangiomas. Correct preoperative diagnosis and initial therapy may therefore be the most important prognostic factors.

** Key words:**Acinic cell carcinoma, Kaplan-Meier analysis, minor salivary glands, prognosis, upper lip.

## Introduction

Acinic cell carcinoma (ACC) is an uncommon malignant tumor of the salivary glands. This tumor usually has low-grade features and a good prognosis (1). The most common location of ACC is the parotid gland, and ACCs of the minor salivary glands (MSGs) are relatively rare. Few studies have reviewed the reported cases of ACC of the MSGs. Kobayashi reported a case of ACC of the MSGs and reviewed the Japanese literature from 1955 to 1998 (2). We report here a case of ACC of the upper lip that was successfully treated. In addition, we review the reported Japanese cases of ACC of the MSGs from 1999 to 2013, and analyze the factors that were associated with prognosis. To our knowledge, this is the first reported study to analyze the prognostic factors for ACC of the MSGs. The results of our analysis suggest a prognostic factor that has not previously been reported.

## Case Report 

**Figure 1:**
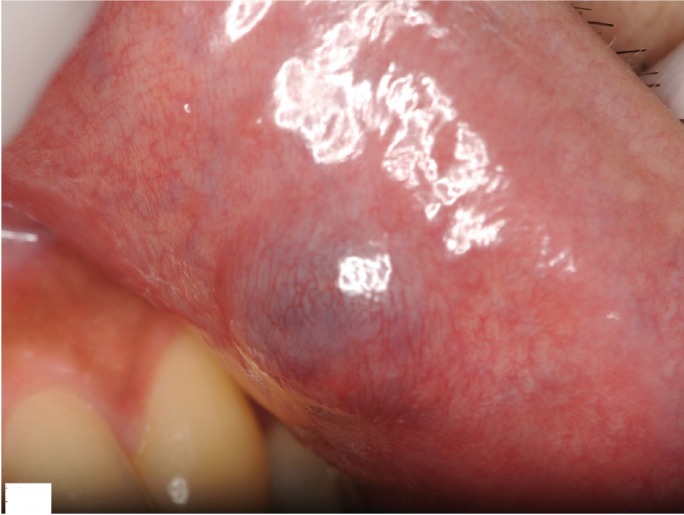
Preoperative view of the upper lip. There is a dark red, elastic, firm mass on the upper lip measuring 12 mm in diameter. The overlying mucosal surface is normal.

**Figure 2:**
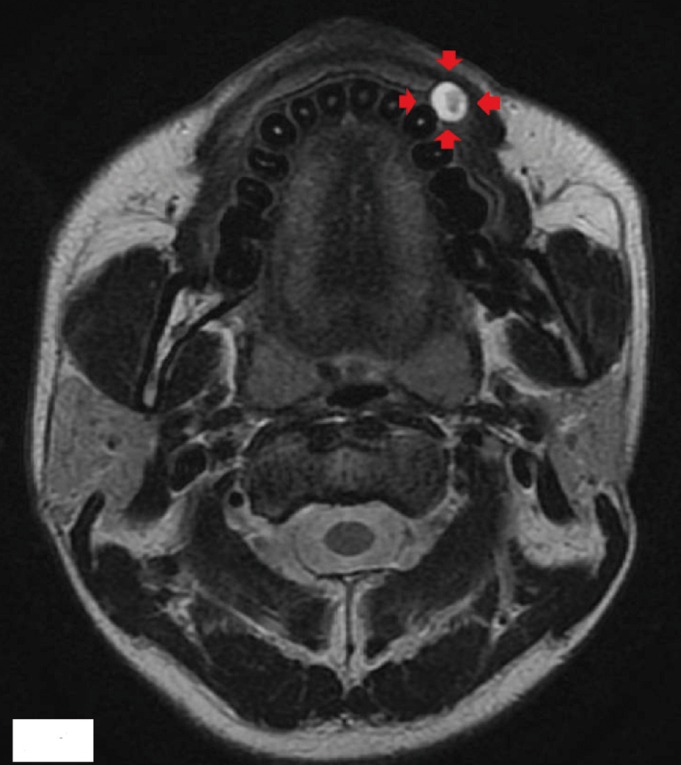
Preoperative magnetic resonance imaging, axial view. There is a 12- × 10- × 10-mm cystic lesion of the upper lip with high signal intensity on T2-weighted images. There is a clearly demarcated border between the lesion and the surrounding tissues.

A 31-year-old male was referred to our clinic because of a nodular mass on his left upper lip (Fig. 1). Examination revealed a dark red, elastic, firm mass on the upper lip measuring 12 mm in diameter. The overlying mucosal surface was normal. Magnetic reso-nance imaging showed a 12- × 10- × 10-mm cystic lesion of the upper lip with clearly demarcated margins (Fig. 2). Enhanced computed tomography did not show enhancement of the mass. The preoperative diagnosis was benign tumor or cyst of the upper lip.

**Figure 3:**
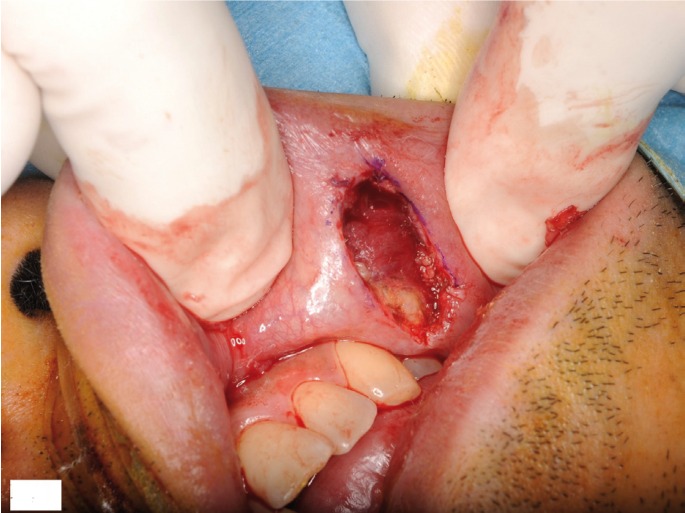
Intraoral view after resection of the tumor.

**Figure 4:**
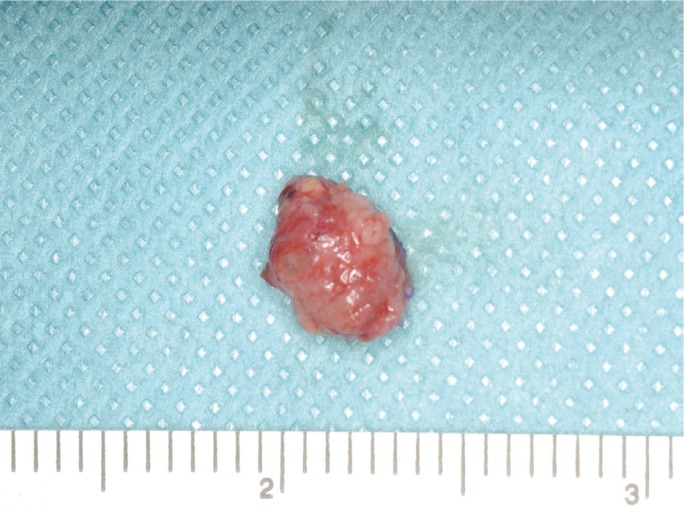
The resected tumor was a well-circumscribed nodule measuring 10 mm in diameter.

The mass was surgically excised along the border with the normal tissues (Fig. 3). The resected tumor was a well-circumscribed nodule measuring 10 mm in diameter (Fig. 4). The cut surface showed a cystic lesion. The pathological diagnosis was ACC with tumor-free surgical margins. The postoperative course was uneventful. No recurrence or metastasis was detected at 13 months postoperatively.

-Pathological findings

**Figure 5:**
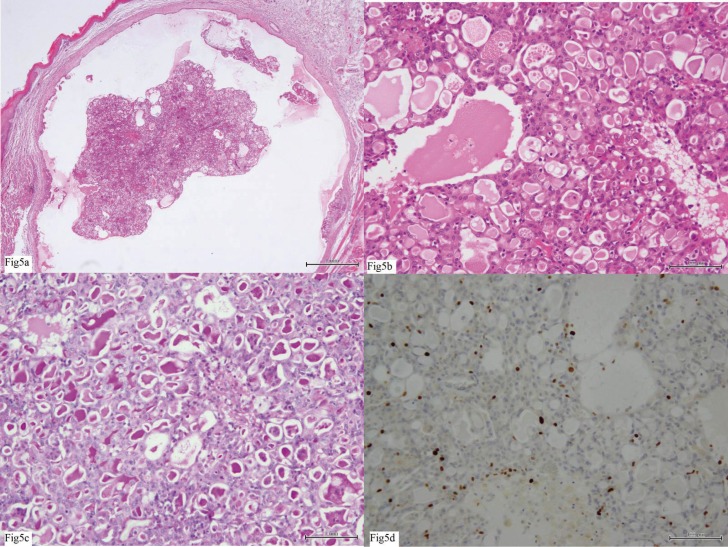
Microscopic examination of the resection specimen. a) The intracystic tumor nodule, which was composed of a small amount of papillary glandular proliferation, appeared to be floating within the cystic cavity (hematoxylin and eosin staining, ×40 magnification). b) The tumor nodule in the cystic cavity showed a thyroid-like follicular pattern, which comprised multiple glands containing homogeneous eosinophilic proteinaceous material (hematoxylin and eosin staining, ×200 magnification). c) The luminal eosinophilic material showed positive periodic acid-Schiff staining after diastase digestion (×200 magnification). d) The percentage of MIB-1-positive tumor cells (Ki-67 labeling index) was approximately 4.0%.

Microscopic examination showed a single cystic papillary tumor surrounded by a thin fibrous capsule, with no evidence of invasion. The intracystic tumor nodule, which contained a small amount of papillary glandular proliferation, appeared to be floating within a cystic cavity (Fig. 5A). The tumor nodule in the cystic cavity showed a thyroid-like follicle pattern that comprised multiple glands containing homogeneous eosinophilic proteinaceous material (Fig. 5B). The luminal eosinophilic material showed positive periodic acid-Schiff staining after diastase digestion (Fig. 5C). The glandular tumor cells were bland and round-to-polygonal vesicular nuclei containing small nucleoli with eosinophilic cytoplasm. Mitotic figures were absent. The percentage of MIB-1-positive tumor cells (Ki-67 labeling index) was approximately 4.0% (Fig. 5D). The nodule was diagnosed as ACC.

## Discussion

Kobayashi reviewed 51 cases of ACC of the MSGs reported in the Japanese literature from 1955 to 1998 (2).  Table 1 summarizes the clinical features and prognosis of the review by Kobayashi (2). Their findings indicated that such cases usually had a good prognosis. Only five patients had cervical lymph node metastasis at the time of diagnosis. Twelve of the 51 cases developed local recurrence, and 9 of the 12 cases of recurrence were controlled by salvage therapy. Regional recurrence was not recognized.

**Table 1:**
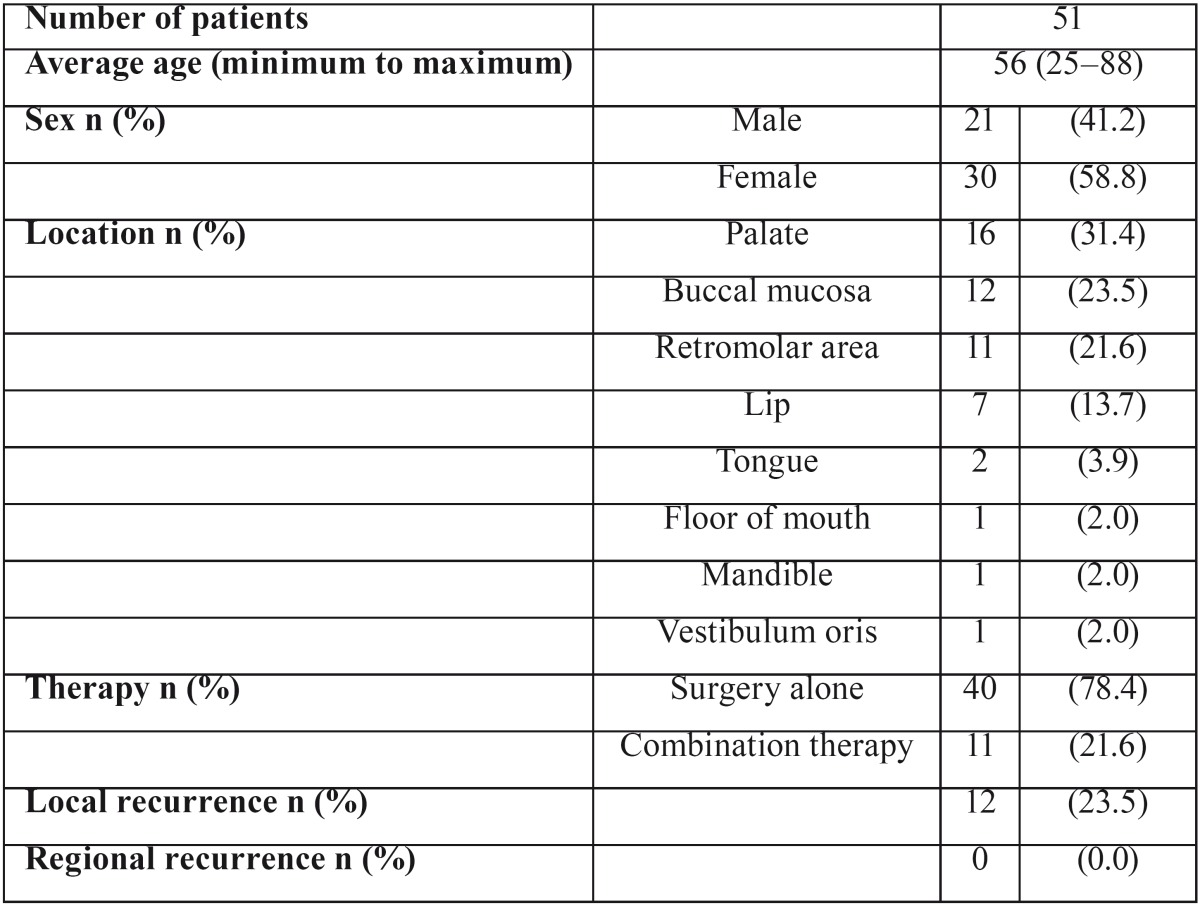
Analyses of 51 cases of ACC in the minor salivary glands in the Japanese literature from 1955 to 1998 by Kobayashi (2).

 Table 2 shows the 21 cases of ACC of the MSGs reported in Japan from 1999 to 2013 (2-18), including our case. All these tumors were staged T1 or T2, and N0. The most frequent site was the buccal mucosa, followed by the lip, gingiva, and palate. The most frequent age range at diagnosis was 50-59 years, followed by 40-49 years and 60-69 years. All patients were alive at the end of the follow-up period, except for one patient with inoperable disease who died while receiving radiotherapy.

**Table 2:**
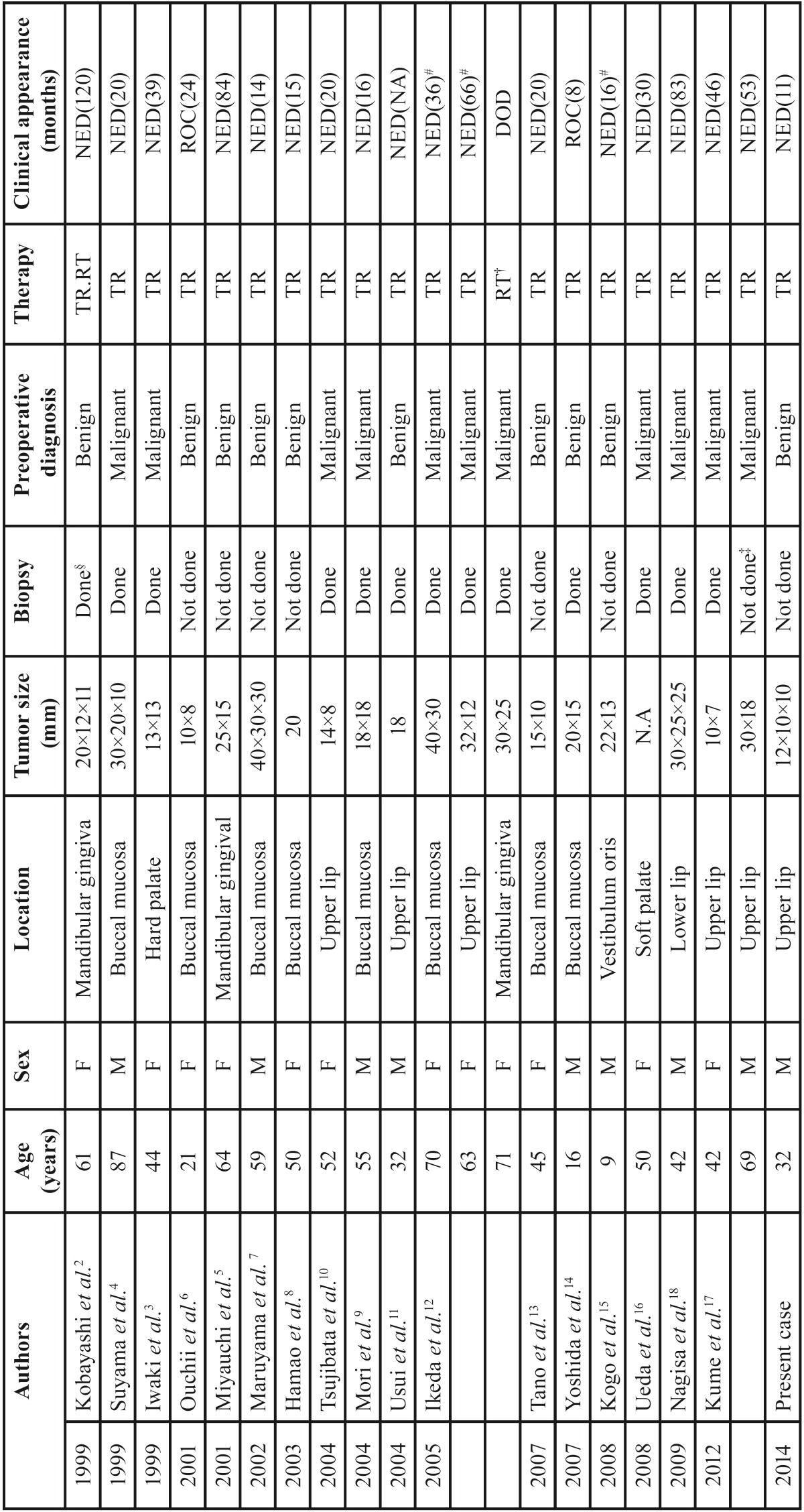
Twenty-one cases of ACC in the minor salivary glands in the Japanese literature from 1999 to 2013.

**Figure 6:**
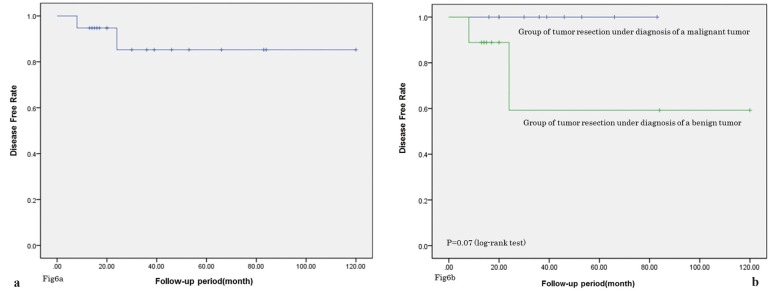
Disease-free survival rate of the follow up period (Kaplan–Meier analysis). a) The 10-year disease-free survival rate was 85.3%. b) The 10-year disease-free survival rate was worse in patients who underwent resection with a preoperative diagnosis of benign tumor than in patients who underwent resection with a preoperative diagnosis of malignant tumor (59.3% versus 100%; *p* = 0.07, log-rank test).

Kaplan–Meier analysis of the 19 cases with detailed follow-up information available showed an overall 10-year disease-free survival rate of 85.3% (Fig. 6A). Recurrence was detected in two cases, both of which underwent salvage therapy. These findings indicate a relatively good prognosis for patients with ACC.

Most ACCs of the MSGs were treated by surgical excision. Radiotherapy was performed in two cases. One patient who under-went surgery received adjuvant radiotherapy, and survived without recurrence (2). One patient with inoperable disease received palliative radiotherapy without surgical excision, and died of their disease (12). Chemotherapy is not administered for ACC of the MSGs in Japan. Chemoradiotherapy was reported to be useful in a few cases (19), but adjuvant chemoradiotherapy may be unnecessary if radical surgery is performed.

Seven of the 21 patients with ACC of the MSGs (including our case) underwent resection with a preoperative diagnosis of benign tumor, without preoperative biopsy. These cases were misdiagnosed as benign lesions such as mucous cysts or hemangiomas (5-8,13,15). Disease-free survival was worse in patients who underwent resection with a preoperative diagnosis of benign tumor than in patients who underwent resection with a preoperative diagnosis of malignant tumor (*p* = 0.07, log-rank test; Fig. 6B). There were no cases of recurrence among patients who underwent resection with a preoperative diagnosis of malignant tumor. The rate of recurrence was higher for ACCs assumed to be benign lesions on a purely clinical basis, or without an accurate preoperative biopsy. Previous review articles that reported on the prognostic factors for ACC included all salivary glands (including the parotid gland). The factors reported to be associated with poor prognosis include short duration of symptoms, incomplete excision, frequent mitoses, focal necrosis, pleomorphism, neural invasion, infiltration, stromal hyalinization, large size, and predominately solid architecture, capsular injury of tumor with surgery (20-24). Especially, incomplete excision and capsular injury of tumor with surgery are well reported as important prognostic factors, however, after all surgical excision as benign tumor will occur the poor prognosis clinically. The results of the current study indicate that correct preoperative diagnosis and initial therapy may be the most important prognostic factors. However, some cases of ACC of the MSGs are difficult to be diagnosed, so a biopsy for the lesion assumed to be benign on a purely clinical basis should be considered. If the excisional biopsy is performed, the preparation for the additional resection with intraoperative frozen section diagnosis may be needed to prevent misdiagnosis.

ACC is characterized by serous acinar cell differentiation, but several cell types and growth patterns have been recognized (25), including acinar, intercalated ductal, vacuolated, clear, non-specific glandular and solid/lobular, microcystic, papillary-cystic, and follicular (19,25-30). Solid and microcystic patterns are the most common, and a follicular pattern (as in our patient) is relatively uncommon (31). Most analyses of relationships between histopathological features and prognosis found that the histopathological features were not a reliable predictor of biological behavior (32).

The proliferation marker Ki-67 has been shown to be one of the most promising predictors of biological behavior. No recurrences of ACC were observed if the Ki-67 labeling index was <5%, whereas most patients with a Ki-67 labeling index >10% had unfavorable outcomes (25,33,34). In our case, the Ki-67 labeling index was 4%, suggesting a good prognosis.

As ACC of the MSGs is relatively infrequent, individual centers in Japan do not experience many cases. This study reviewed all the reported Japanese cases to date, but a multicenter study with multivariate analysis may be needed to further elucidate the prognostic factors for ACC of the MSGs.

We reported a case of ACC of the upper lip with a good outcome, and analyzed the prognostic factors for ACC of the MSGs among the reported Japanese cases from 1999 to 2013. Our findings suggest that correct preoperative diagnosis and initial therapy may be the most important prognostic factors. Some cases of ACC of the MSGs may be misdiagnosed as benign lesions. ACC of the MSGs should therefore be diagnosed with the utmost care and attention.
